# Chimpanzees prioritise social information over pre-existing behaviours in a group context but not in dyads

**DOI:** 10.1007/s10071-018-1178-y

**Published:** 2018-03-24

**Authors:** Stuart K. Watson, Susan P. Lambeth, Steven J. Schapiro, Andrew Whiten

**Affiliations:** 1 Scottish Primate Research Group, Centre for Social Learning and Cognitive Evolution, School of Psychology and Neuroscience, University of St Andrews, St Andrews, UK; 2 Department of Veterinary Sciences, National Center for Chimpanzee Care, Michale E. Keeling Center for Comparative Medicine and Research, The University of Texas MD Anderson Cancer Center, Bastrop, TX USA; 3 Department of Experimental Medicine, University of Copenhagen, Copenhagen, Denmark

**Keywords:** Chimpanzee, Culture, Conformity, Social learning

## Abstract

How animal communities arrive at homogeneous behavioural preferences is a central question for studies of cultural evolution. Here, we investigated whether chimpanzees (*Pan troglodytes*) would relinquish a pre-existing behaviour to adopt an alternative demonstrated by an overwhelming majority of group mates; in other words, whether chimpanzees behave in a conformist manner. In each of five groups of chimpanzees (*N* = 37), one individual was trained on one method of opening a two-action puzzle box to obtain food, while the remaining individuals learned the alternative method. Over 5 h of open access to the apparatus in a group context, it was found that 4/5 ‘minority’ individuals explored the majority method and three of these used this new method in the majority of trials. Those that switched did so after observing only a small subset of their group, thereby not matching conventional definitions of conformity. In a further ‘Dyad’ condition, six pairs of chimpanzees were trained on alternative methods and then given access to the task together. Only one of these individuals ever switched method. The number of observations that individuals in the minority and Dyad individuals made of their untrained method was not found to influence whether or not they themselves switched to use it. In a final ‘Asocial’ condition, individuals (*N* = 10) did not receive social information and did not deviate from their first-learned method. We argue that these results demonstrate an important influence of social context upon prioritisation of social information over pre-existing methods, which can result in group homogeneity of behaviour.

**Electronic supplementary material:**

The online version of this article (10.1007/s10071-018-1178-y) contains supplementary material, which is available to authorized users.

## Introduction

Culture emerges and is maintained by a suite of social learning mechanisms and biases that govern how social information is transmitted and when individuals choose to prioritise social information over pre-existing methods. One such proposed bias is conformity, defined as foregoing a pre-existing behaviour in favour of adopting one demonstrated by a majority of conspecifics (Haun and Tomasello 2011; Whiten and Van Schaik 2007). This represents an important contrast with ‘conformist transmission’, which refers to the tendency for *naïve* individuals to disproportionately copy the behaviour of a majority (Boyd and Richerson 1988; Van Leeuwen and Haun 2013). Conformity is a well-established bias in humans, occurring across varied cultures and age groups (Bond and Smith 1996). However, whether non-human species exhibit conformist behaviour is a topic of recent debate (Claidière and Whiten 2012). While experimental evidence has been offered for conformist behaviour in nine-spined stickleback fish (Pike and Laland 2010), great tits (Aplin et al. 2015b), vervet monkeys (van de Waal et al. 2013) and chimpanzees (Whiten et al. 2005), each of these examples has been critiqued for not systematically ruling out alternative explanations (Acerbi et al. 2016; Haun et al. 2013; Van Leeuwen and Haun 2013, 2014; Van Leeuwen et al. 2015). For example, it has been suggested that so-called conformist individuals could have copied the most frequently observed behaviour rather than the behaviour of a majority of individuals (van Leeuwen et al. 2015; c.f. Aplin et al. 2015a) or simply copied one or more individuals at random, which could lead to the same effect of acting like a majority in the group (Acerbi et al. 2016; c.f. Smaldino et al. 2017). Three further studies have experimentally investigated whether our closest extant relatives, chimpanzees, behave in a conformist way, none of which found evidence to support this claim (Haun et al. 2014; Vale et al. 2017; Van Leeuwen et al. 2013). However, each of these studies had a confound that may explain these results. Van Leeuwen et al. (2013) used minority *subgroups* rather than lone minorities faced with an overwhelming majority, which we know is critical in motivating conformity effects in humans (Asch 1951). In Haun et al. (2014) and Vale et al. (2017), subjects had prior experience that the majority method was either ineffective (no reward given for this method during training) or associated with unpalatable food rewards, respectively, before being exposed to the majority method. This introduced an asymmetry in the likely payoffs of each method, which chimpanzees are sensitive to when making social learning decisions (Van Leeuwen et al. 2013) and may therefore explain the lack of conformist behaviour. Human conformity can be sufficiently strong to override such negative valence associated with the majority method, but only in a minority of humans tested (Asch 1951; Bond and Smith 1996), and this is not a critical part of the definition of conformity adopted in our opening paragraph. It therefore remains a possibility that conformity is more readily expressed by animals in contexts that lack this element, and where there is unanimity in the observed responses, a hypothesis we thus explore in the present study.

Consistent with this hypothesis, Luncz and Boesch (2014) offer evidence that, when migrating, wild female chimpanzees conform to the new tool-use norms of the community they transfer to. Similar effects have been reported in migratory vervet monkeys (van de Waal et al. 2013), great tits (Aplin et al. 2015a, b) and possibly meerkats (Thornton et al. 2010). Furthermore, chimpanzees who explored alternative methods after their group was seeded with a method of solving a puzzle box nevertheless converged on homogeneity of behaviour, a disposition the authors suggest may have been conformity to the majority preference (Whiten et al. 2005). However, this has been suggested to be explicable by reversion to an individual’s first-learned method, a hypothesis that cannot be rejected without further experimental testing (Van Leeuwen and Haun 2013).

**Fig. 1:**
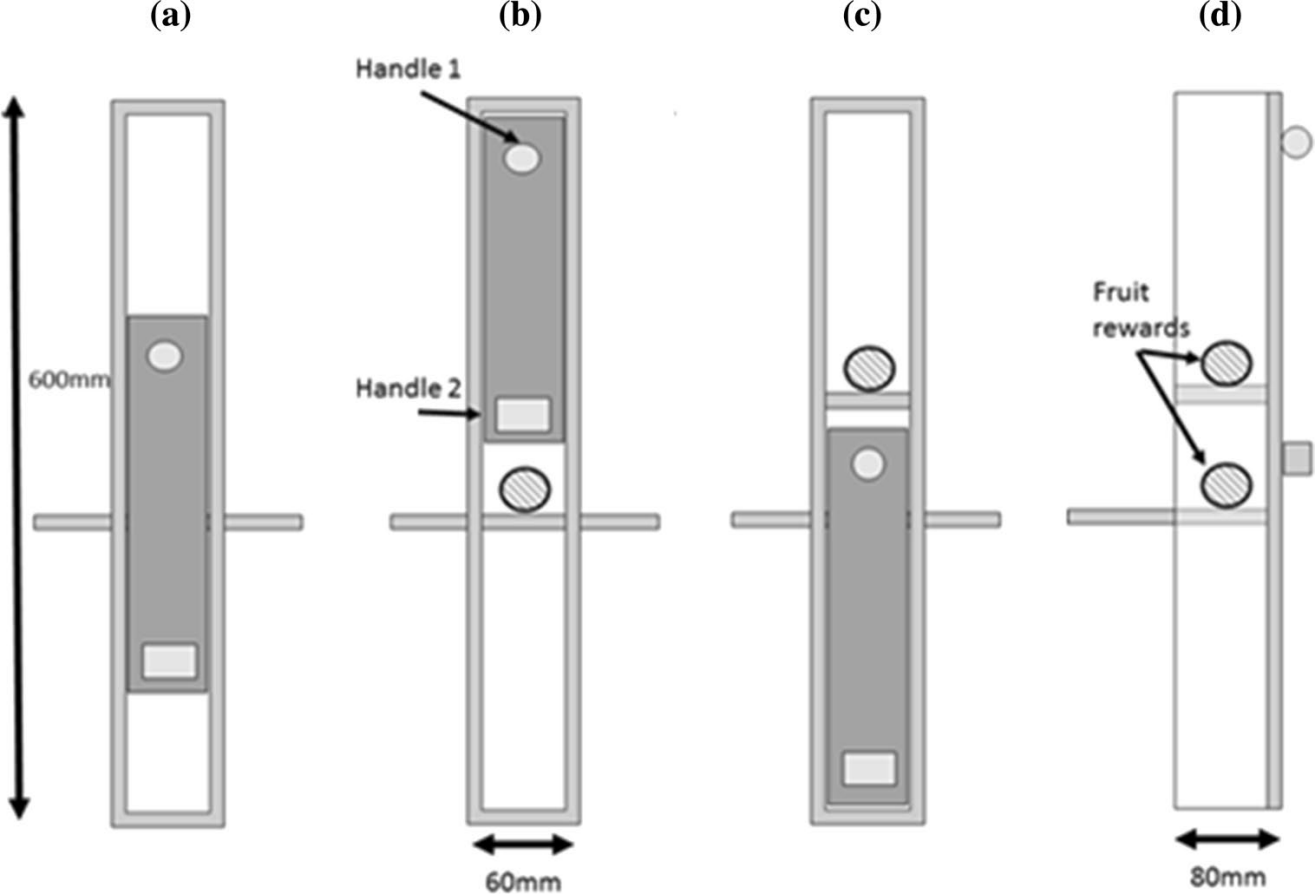
The box could be opened to reveal a food reward by either sliding the door entirely upwards (**b**) or entirely downwards (**c**). The resting position on presentation is shown in (**a**). The side profile is shown in (**d**). Upon a completed opening, the door locked so as to restrict access to the alternative reward. The anchor platform was attached to a trolley with vice clamps to stabilise the apparatus

In the present study, we investigated whether chimpanzees proficient in a pre-existing minority method (minority individual/s = ‘MIN-I’) of opening a puzzle box (Fig. 1), and who were naïve to alternative methods, would converge on the different behaviour demonstrated in a group context by an overwhelming majority of group mates (majority individual/s = ‘MAJ-I’). This open-diffusion ‘Group’ condition lasted for a total of 5 h. We predicted that if subjects were to demonstrate conformist behaviour, they would maintain their trained behaviour until they had observed a majority of their group mates demonstrate, at which point they would converge on this majority method. In order to explore whether frequency of observations (as opposed to number of individuals observed, Van Leeuwen et al. 2015), asocial exploration or random copying (Acerbi et al. 2016) might account for changes in behavioural preference in the Group condition, we introduced two further conditions. In a ‘Dyad’ condition, we paired individuals who were trained on alternative methods of opening the apparatus and observed whether either would converge on the behaviour of their partner over 1 h of access to the apparatus. We predicted that if frequency of observed behaviour or random copying can elicit behaviour switching, levels of switching should be similar between the Dyad and Group condition. In an ‘Asocial’ condition, a single individual was trained on one method to explore whether they would switch to an alternative method without social information during 30 min of unrestricted access to the task.

## Methods

### Participants and study site

Participants were 59 chimpanzees (Group condition MIN-I *N* = 5: all female, Group condition MAJ-I: *N* = 32, 17 females, Dyad condition *N* = 12: nine females, Asocial condition *N* = 10: four females) housed at the National Center for Chimpanzee Care located at the Michale E. Keeling Center for Comparative Medicine and Research of the University of Texas MD Anderson Cancer Center in Bastrop (UTMDACC), Texas (see Table 1 for full demographic information). The size of groups used in the Group condition varied between 5 and 9 individuals. Data were collected between April and August 2016. All individuals were naïve to the apparatus prior to training except two (BER, KUD) in the Dyad condition who had previously participated in Watson et al. (2017). These individuals were used to provide sufficient partners for the Dyad condition, but were excluded from all analyses. Ethical approval for this study was granted by the School of Psychology and Neuroscience at the University of St Andrews and the IACUC of UTMDACC, adhering to all the legal requirements of US law and the American Society of Primatologists’ principles for the ethical treatment of non-human primates. All subjects voluntarily participated in the testing procedures.

**Table 1:** Demographic information for each participating individual

ID	Condition	Sex	Age	Direction of trained method
HAN	**Group** (**1**)	**F**	**26**	**U**
COR	Group (1)	M	45	D
APR	Group (1)	F	36	D
SIN	Group (1)	F	17	D
COC	Group (1)	F	32	D
**DAH**	**Group** (**2**)	**F**	**31**	**D**
EES	Group (2)	F	30	U
KAL	Group (2)	M	35	U
KUH	Group (2)	M	36	U
MAI	Group (2)	F	34	U
OKI	Group (2)	F	32	U
PAH	Group (2)	F	30	U
STA	Group (2)	M	32	U
BAH	Group (3)	M	34	U
CAT	Group (3)	F	37	U
EHS	Group (3)	M	21	U
ENI	Group (3)	F	31	U
**IDA**	**Group** (**3**)	**F**	**32**	**D**
KEH	Group (3)	M	34	U
TAN	Group (3)	F	48	U
TOT	Group (3)	M	22	U
GRA	Group (4)	F	24	U
**JOS**	**Group** (**4**)	**F**	**26**	**D**
MAH	Group (4)	F	26	U
PIM	Group (4)	M	24	U
PIP	Group (4)	M	22	U
SHA	Group (4)	M	26	U
SUH	Group (4)	F	26	U
TAM	Group (4)	M	26	U
TOH	Group (4)	M	27	U
**AHN**	**Group** (**5**)	**F**	**22**	**U**
CHU	Group (5)	F	36	D
GAY	Group (5)	M	25	D
HUG	Group (5)	F	19	D
HUH	Group (5)	M	33	D
KIA	Group (5)	F	29	D
SAH	Group (5)	F	26	D
AJA	Asocial	M	39	D
AUS	Asocial	M	25	D
BET	Asocial	F	42	D
CHES	Asocial	M	21	U
GIS	Asocial	M	33	U
JOE	Asocial	M	45	D
KAM	Asocial	F	26	U
MIS	Asocial	F	30	U
SIM	Asocial	M	46	U
WOT	Asocial	F	34	D
BER	Dyad	F	39	D
BIL	Dyad	M	23	D
CHEC	Dyad	F	35	D
DOD	Dyad	F	34	U
JES	Dyad	F	24	U
KUD	Dyad	M	35	U
LUL	Dyad	F	35	D
NAH	Dyad	F	35	U
NOW	Dyad	M	33	U
PEP	Dyad	F	50	U
PRI	Dyad	F	49	D
SAB	Dyad	F	49	D

Bold indicates individual was MIN-I

*M* male, *F* female, *C* captive, *W* wild

### Apparatus

This study employed a two-action, sliding-door puzzle box (Fig. 1), a design that has been successfully used to examine social learning in previous work (Aplin et al. 2015a, b; Hopper et al. 2008; Kendal et al. 2015; Watson et al. 2017). Some of the individuals at the study site had previously been exposed to horizontally oriented puzzle boxes (Hopper et al. 2008; Kendal et al. 2015), and so, in order to minimise directional bias from prior experiments when sampling the same individuals, we gave the apparatus a vertical orientation. All training and experimental sessions were recorded using a Panasonic HC-X920 video camera. Videos were directly transferred in high-definition ‘.mts’ format to an ASUS laptop computer. All videos were coded using BORIS (Behavioural Observation Research Interactive Software) version 2.05 (2015).

### Experimental procedure

All three conditions consisted of a training phase followed by open access. In the Group condition, the single MIN-I for each of five groups voluntarily separated from their group and learned to open the apparatus door by sliding it either up or down (counterbalanced across groups). At least 80% of the remaining group members (MAJ-I) were trained on the alternative method. This was followed by 5 h (except Group 3, which had four) of open access to the apparatus, 1 h on each consecutive weekday. In order to explore whether behavioural changes persisted without the presence of their group, after open access finished we retested MIN-I individuals in two 20-min ‘solo’ sessions. Finally, during the second week after finishing the open-access phase, we carried out one final hour of open-access testing. This was to determine whether any observed changes in behavioural preference were stable over time. The Dyad condition followed the same procedure, using just two chimpanzees and 1 h of open access. This amount of time was advised by care staff as being a realistic period in which any two given individuals would be comfortable being separated from their group as a pair, while also providing ample opportunities for observation (in the case of the dyad condition) and access to the puzzle box. The Asocial condition used individual chimpanzees, which were each provided with 30 min of open access to the apparatus. This was a length of time advised by care staff as a realistic period for which most individuals would be comfortable being separated from their group, while also providing the individual with opportunity for a large number of trials. The reward for successfully opening the box in all conditions was a single grape. A detailed description of the methods used for training and each condition can be found below.

### Group condition

The group condition comprised four stages: (1) training, (2) open access, (3) solo sessions and (4) a final open-access session, as detailed below.

### Stage 1: Training

In each group, a single individual was selected as the MIN-I who was trained on one method of opening the apparatus (either ‘up’ or ‘down’). Previous work with the apparatus established that chimpanzees do not have a strong directional bias towards either option (Watson et al. 2017), but nevertheless we counterbalanced trained methods across groups (three MIN-I trained on ‘up’, two on ‘down’, see Table 1). MIN-I were chosen based on the advice of care staff who have known the animals for 5 + years, selecting in each case a female individual who was of medium-to-low social rank so that they would be able to gain access to the task but would not monopolise it. The rationale for this was that observational accounts of wild chimpanzees exhibiting conformist behaviour involve females migrating to a new group, which they typically enter at the lower end of the hierarchy (Luncz and Boesch 2014). All other members of the group were designated as MAJ-I. As many of these individuals as possible were trained on the alternative method to that which the MIN-I of their group was trained on. Four individuals in the Group condition were not willing to participate in training at all and did not engage with the task (though they were physically present) during later sessions. This was the case for no more than one individual per group, still leaving an effective majority of individuals trained on the majority method.

The training process for method learning was facilitated by leaving the door of the puzzle box halfway open so that the trainee could see the reward and access it easily. On subsequent trials, the puzzle box door was left increasingly closed so that the trainee had to move it to get the grape. This continued until the trainee was able to open it from a fully closed position. Models were considered to be ‘trained’ once they completed a total of 30 sequential uses of the trained method without deviation. No individuals deviated from the trained method during training, meaning that each individual completed exactly 30 training trials. The alternative direction was not blocked in any way. This number of trials was chosen as it was thought to be sufficient to instil a strong behavioural preference in the trained individual, making deviation unlikely without potent external motivating factors (Hopper et al. 2011; Hrubesch et al. 2009; Marshall-Pescini and Whiten 2008). With just two exceptions, all individuals in all conditions were trained while separated from the rest of their group. The two individuals who were not comfortable being separated from their group were therefore trained while in each other’s company.

### Stage 2: Open access

Stage 2 consisted of 5 h of unrestricted access to the apparatus in a group context, during which time any individual was able to approach and manipulate the apparatus or observe others doing so. Access was divided into single hour-long testing sessions which, when possible, were carried out on consecutive days (Monday to Friday). One group received only 4 h of open access as it was not possible to test on the fifth day. The number of trials carried out by each MIN-I and MAJ-I as a whole is shown in Table 3. For each trial of Stage 2, the apparatus was baited with a single grape in each reward chamber and pushed towards the mesh of an enclosure, facing forwards, and held there until an individual approached and successfully opened the door. After an individual retrieved a reward, the apparatus was withdrawn by 1 m, the door was reset to the central position, and the reward chambers were both re-baited. When re-setting the door, the apparatus was covered with a cloth to avoid directional cues from the experimenter. If the door was partially opened by an individual and no further interaction occurred for 10 s, the apparatus was withdrawn and reset as described above. Any individuals within 3 m whose heads were oriented towards the puzzle box and did not have their view obviously obstructed were recorded as having observed the trial.

### Stage 3: Solo sessions

At 3–5 days after completion of Stage 2, MIN-I were separated from their group and given 20 min of access to the puzzle box (‘Solo Session 1’). This happened again 7–10 days following Solo Session 1 (‘Solo Session 2’). The purpose of these sessions was to discover whether any behavioural changes in MIN-I were maintained in the absence of observers.

### Stage 4: Final open-access session

Between 4 and 8 days after Stage 3, the entire group was given a final open-access session with the apparatus, lasting 1 h. This followed the same protocol as Stage 2. The purpose of this was to determine whether any behavioural changes in MIN-I were persistent over time.

### Dyad condition

For each of the six dyads (*N* = 12), two individuals were selected from the same group. Individuals were selected based on the advice of care staff regarding which individuals were likely to be comfortable sharing a room with each other for an hour. Once selected, each individual in the dyad was individually trained on alternative methods (‘up’ and ‘down’) of opening the apparatus. The procedure for training followed the same protocol as for MIN-I in the Group condition. Two individuals in the Dyad condition had prior exposure to the task and so were not included for analysis (but their partner was).

The day after training had taken place for a dyad, the two individuals were separated from their group, as a pair, for 1 h. During this hour, unrestricted access to the apparatus was provided. This followed the same procedure as Stage 2 of the Group condition. This open-access phase of the Dyad condition was limited to 1 h, as the feasibility of getting two specific individuals alone together on five consecutive days was expected to be low. Furthermore, based on the advice of care staff, 1 h was judged to be a length of time in which two individuals would reliably participate in the task before becoming noticeably motivated to return to their group. Secondly, prior work (Watson et al. 2017) using the same puzzle box suggested the box could be opened and re-baited at a rate of roughly two trials per min. Given the rapid onset of conformist behaviour in previous studies (Aplin et al. 2015b; Pike and Laland 2010; van de Waal et al. 2013) and indeed the fact that all MIN-I who switched did so within their first five trials, this was judged to be an adequate amount of exposure to the task for behavioural switching to manifest.

### Asocial condition

Individuals (*N* = 10) in the Asocial condition received the same training as those in the Dyad condition. The next day, subjects received 30 min of unrestricted access to the apparatus while alone, having never observed another individual interact with it. The purpose of this condition was to determine how frequently chimpanzees would explore the untrained method when not provided with social information about it, to determine whether this is sufficient to explain switching patterns in the Group and Dyad conditions. Sessions were limited to 30 min primarily to minimise the amount of time that individuals spent alone and separated from group mates. Furthermore, 30 min allowed for a potential of ~ 60 trials per individual, which was judged to be sufficient access to the task for motivated individuals to explore an alternative method.

### Statistical analyses

We used generalised linear mixed models (GLMMs), using R packages ‘lme4’ and ‘MuMin’ (Bates et al. 2016; MuMIn 2016) with a binomial error structure and a logit link function to determine whether ‘Condition’ (MIN-I vs. MAJ-I, MIN-I vs. Dyad and Dyad vs. Asocial) had a significant effect upon our response variable: a binary indicator of whether an individual used their trained or untrained method on any given trial. When comparing MIN-I with MAJ-I and MIN-I with Dyad conditions, we also fit as a fixed effect the number of demonstrations an individual had observed of their untrained method on each trial.

In each case, we fitted a ‘full’ model containing all fixed effects. Any non-significant effects were dropped from the model to create a ‘final’ model, which we then compared with the ‘null’ model (no fixed effects) using a likelihood ratio test to determine whether either was a significantly better fit for the data. Because each individual contributed multiple data points, we fitted Individual as a random factor in all models. In the first analysis, comparing MIN-I and MAJ-I responses, test session (h from 1 to 5) was also fitted as a random effect. For each final model, we also calculated a marginal *R*^2^ value, which describes the proportion of variance explained by the fixed effects (Nakagawa and Schielzeth 2013). We used the R package ‘rptR’ (Schielzeth and Nakagawa 2011) to estimate whether there was a significant effect of repeatability (where *H*_0_ is *R* = 0) between the proportion of trials in which MIN-I used their untrained method firstly in Stages 2 and 3 and then in Stages 2 and 4. All analyses were conducted in R v.3.2.3 (R Development Core Team 2016 with R Studio v.0.99.491 (R Studio Team 2015).

Inter-observer reliability was carried out with an independent observer on the method (‘up’ or ‘down’) used in thirty 30-s video clips of individuals opening the apparatus, as well as which individuals observed those demonstrations, with 100% agreement. The datasets analysed during the current study are available in the open science framework repository and can be accessed at: https://osf.io/seq8b/.

## Results

### Analysis 1: MIN-I versus MAJ-I

**Fig. 2:**
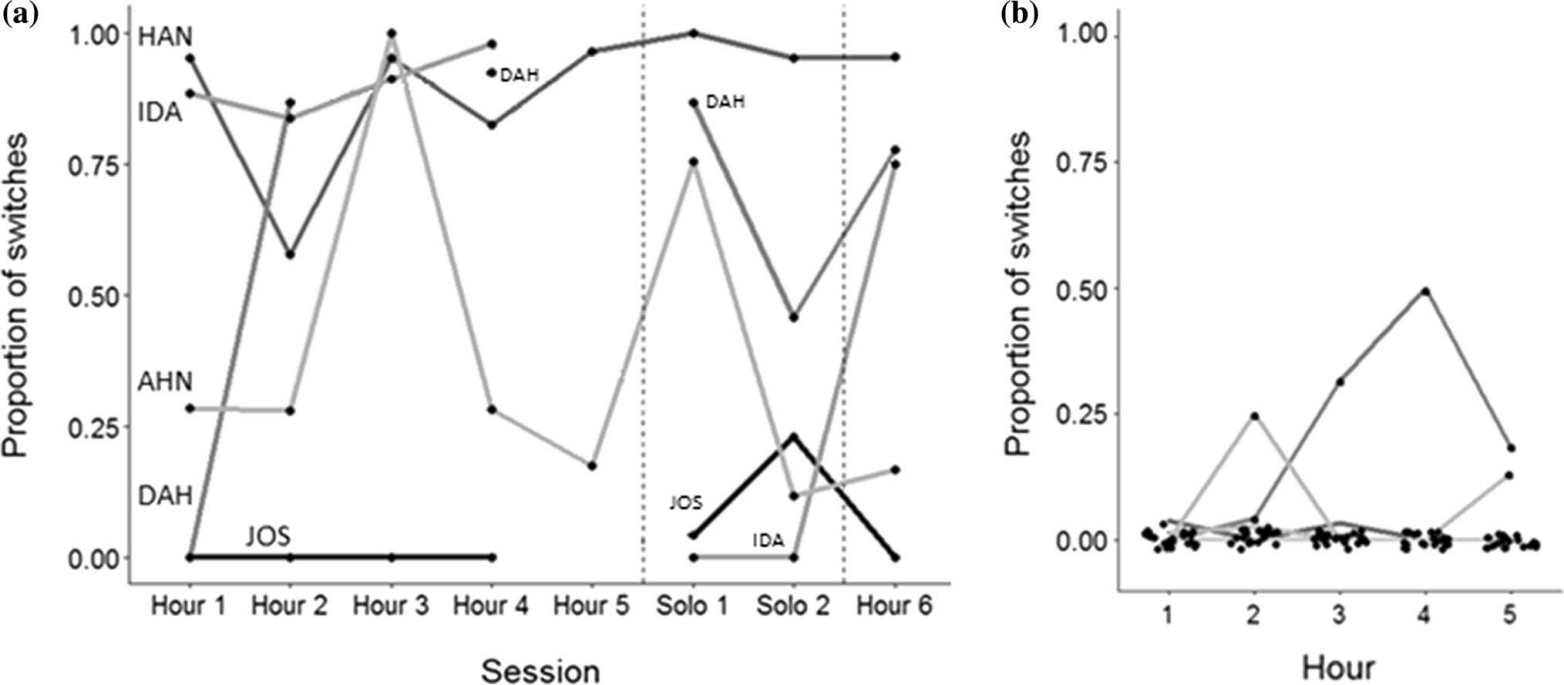
Proportion of trials in which an individual used their untrained method in each hour. **a** MIN-I (*N* = 5) across all stages, **b** MAJ-I (*N* = 23) in Stage 2. Each line represents an individual. Dashed vertical lines serve as a visual aid for contrasting solo and group stages. Points in **b** are jittered to avoid overlapping. Not all individuals participated in all sessions

In the Group condition, four out of five MIN-I learned the majority method, all of whom did so after observing at least one MAJ-I, but before observing the majority of their group (Table 2). Three of these individuals used their untrained method on the majority of trials in each test session, a response already apparent in the first 1-h session, but which was sustained also in the later sessions (Fig. 2, Table 3). All individuals in both MIN-I and MAJ-I had multiple observations of their untrained method by the end of the fifth hour of testing (MIN-I: median = 104, minimum = 72, maximum = 117, MAJ-I: median = 31, minimum = 2, maximum = 109). All individuals in both conditions participated in at least one trial by the end of the fifth hour of testing (MIN-I: median = 124, minimum = 37, maximum = 339, MAJ-I: median = 80, minimum = 1, maximum = 289). With the full model, we found a significant effect of Condition, but not frequency of observations of an individual’s untrained method, on switching behaviour. Consequently, we dropped frequency of observations as a fixed effect in the final model (Table 4). This final model was found to be a significantly better fit for the data than the null model (likelihood ratio test: *X*^2^ = 8.333, *df* = 1, *p* = 0.003, Table S1). With the final model, we estimated that the probability that MIN-I would switch to their untrained method on any given trial was 0.54 (95% CI 0.262, 0.791), whereas the probability of MAJ-I switching methods was less than 0.001 (95% CI 0.000, 0.002). The proportion of variance explained by the fixed effects in the final model was *R*^2^ = 0.515. Finally, we examined whether the number of MAJ-I, or number of observations of MAJ-I, that MIN-I had observed using their untrained method influenced their choice of method on any given trial. Neither number of observations nor number of individuals was found to have a significant effect on method choice (Table 4).

**Table 2:** Trial number on which MIN-I first switched to untrained method, number of individuals they had observed using this method by that point, and total number of group mates (minus MIN-I)

ID	Trial of first switch	Hour of first switch	Number MAJ-I seen at the time of switch	No. group mates that participated	Total group size
JOS	No switch	No switch	No switch (6 seen)	6	8
HAN	2	1	1	4	4
DAH	4	2	3	6	7
IDA	4	1	2	6	7
AHN	2	1	2	6	6

**Table 3:** Number of trials MIN-I and corresponding MAJ-I used their trained and untrained methods in each test session

ID	Method	Hour 1	Hour 2	Hour 3	Hour 4	Hour 5	Solo 1	Solo 2	Hour 6	Total
JOS	Trained	14	11	3	9	0	46	37	35	155
	Untrained	0	0	0	0	0	2	11	0	13
	Total	14	11	3	9	0	48	48	35	168
MAJ-I	Trained	79	76	57	91	94	–	–	85	482
	Untrained	1	1	14	3	2	–	–	4	25
	Total	80	77	71	94	96	–	–	89	507
HAN	Trained	4	25	4	10	2	0	2	2	49
	Untrained	79	34	80	47	54	40	45	42	421
	Total	83	59	84	57	56	40	47	44	470
MAJ-I	Trained	30	87	64	50	57	–	–	76	364
	Untrained	0	0	0	0	0	–	–	0	0
	Total	30	87	64	50	57	–	–	76	364
DAH	Trained	2	6	0	2	0	7	25	14	56
	Untrained	0	39	0	24	0	45	21	49	178
	Total	2	45	0	26	0	52	46	63	234
MAJ-I	Trained	70	69	119	86	123	–	–	57	524
	Untrained	0	0	0	0	0	–	–	0	0
	Total	70	69	119	86	123	–	–	57	524
IDA	Trained	3	9	7	1	–	63	59	7	149
	Untrained	23	46	73	44	–	0	0	35	221
	Total	26	55	80	45	–	63	59	42	370
MAJ-I	Trained	88	63	43	74	–	–	–	78	346
	Untrained	0	1	0	0	–	–	–	0	1
	Total	88	64	43	74	–	–	–	78	347
AHN	Trained	5	18	0	23	19	14	52	6	137
	Untrained	2	7	37	9	4	43	7	2	111
	Total	7	25	37	32	23	57	59	8	176
MAJ-I	Trained	111	66	79	85	84	–	–	90	515
	Untrained	1	1	0	1	4	–	–	0	7
	Total	112	67	79	86	88	–	–	90	522

Dashes indicate that no testing took place

**Table 4:** Summary outputs for each final model tested

Conditions compared	Fixed effect	Beta	SE	Lower 95%	Upper 95% CI	*Z*	*p*
MIN-I versus MAJ-I	Intercept	− 10.778	2.215	− 15.12	− 6.436	− 4.866	–
	Condition	10.926	2.82	5.399	16.452	3.875	< 0.001
Within MIN-I	Intercept	1.712	1.715	− 1.649	5.074	0.998	0.318
	*N* individuals seen	− 0.089	0.258	− 0.594	0.417	− 0.343	0.732
	*N* trials seen	− 0.014	0.009	0.033	0.004	− 1.484	0.138
Dyad	Intercept	11.457	3.632	− 18.475	− 4.338	− 3.155	–
Versus asocial	Condition	0.377	3.469	− 6.422	7.176	0.109	0.913
MIN-I versus Dyad	Intercept	− 8.562	1.897	12.279	4.844	− 4.514	–
	Condition	8.948	2.242	4.554	13.342	3.991	< 0.001

It was found that there was non-significant repeatability between Stages 2 and 3 (*R* = 0.309, 95% CI 0, 0.852, *p* = 0.322), suggesting that MIN-I behaved differently depending on whether they were in a group context or by themselves (Fig. 2a, Table 3). There was a significant effect of repeatability between Stages 2 and 4 (*R* = 0.862, 95% CI 0.183, 0.981, *p* = 0.007), demonstrating that switching behaviour was persistent over time. The confidence intervals for these estimates are very wide due to the small sample size used to calculate them, so should be interpreted with caution (Zou 2012).

### Analysis 2: Dyad versus Asocial conditions

Of all individuals in the Dyad condition (*N* = 12), only one explored the method demonstrated by their partner (2/65 trials). Only one individual in the Asocial condition (*N* = 10) discovered the untrained method and used it on only a single trial out of 10. Individuals in the Asocial condition had a median of 55 trials (range 10–80), while individuals in the Dyad condition had a median of 64 trials (range 6–100). In the full model comparing Dyad and Asocial individuals, there was no significant effect of Condition (Table 4). Consequently, we dropped this fixed effect and use the null model as our final model, from which the full model did not significantly differ (log-likelihood ratio test: *X*^2^ = 0.012, *df* = 1, *p* = 9135). From this final model, we calculated that the probability with which any individual would switch to their untrained method on any given trial was less than 0.001 (95% CI 0.000, 0.003).

### Analysis 3: MIN-I versus Dyad conditions

**Fig. 3:**
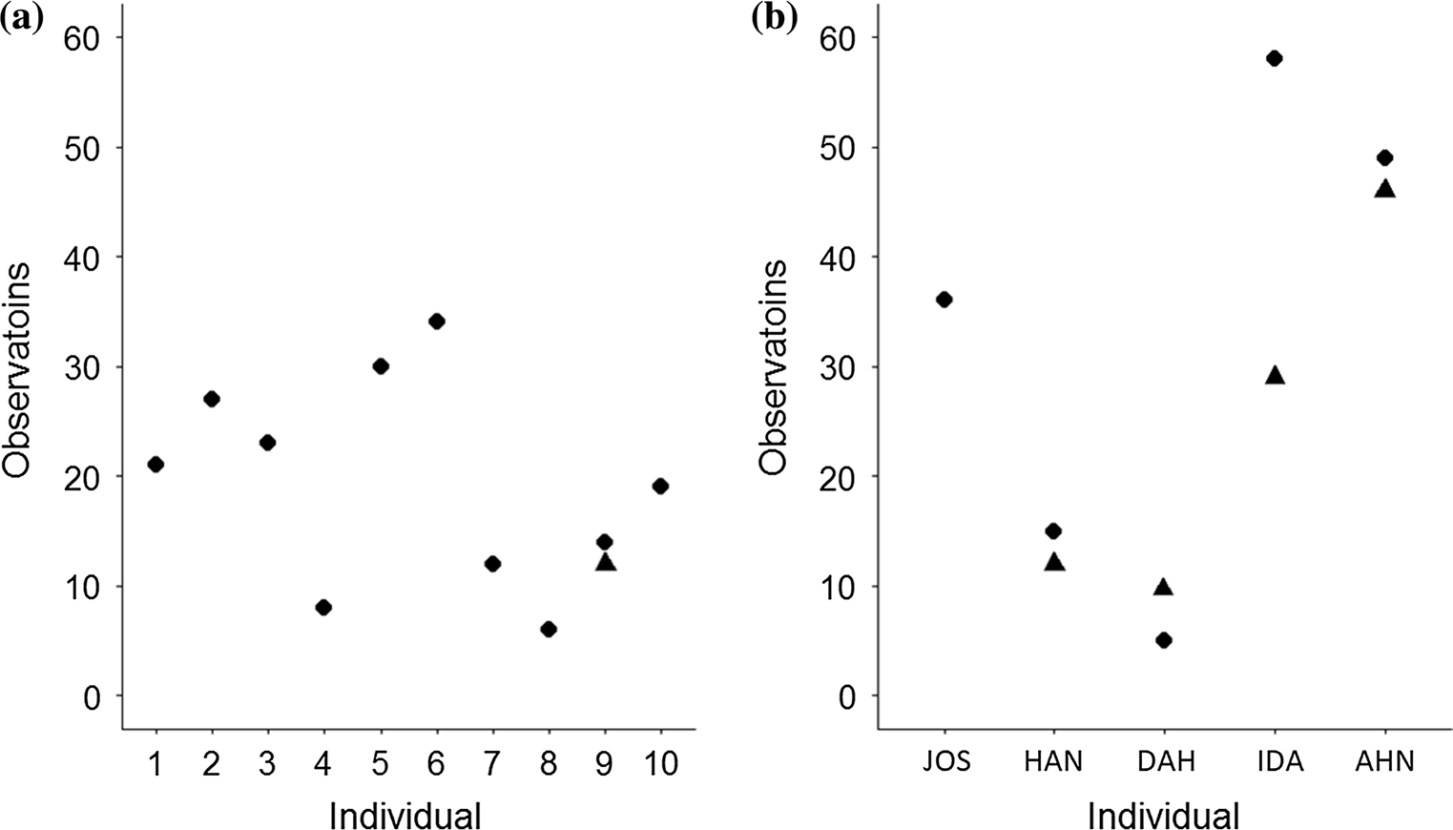
Number of times individuals in the Dyad condition (**a**) and MIN-I (**b**) observed individuals using their untrained method. Circles represent total number of observations in first hour of testing. Triangles represent number of observations by the time of an individual’s first switching event

The median number of observations in Dyad and MIN-I conditions was 18 (range 2–34) and 29 (range 5–46), respectively, and the two groups did not differ significantly (*n*_Dyad_ = 10, *n*_minority_ = 5, *U* = 18, *p* = 0.439, two-tailed) in their number of observations at the time of switching nor their total number of observations (*n*_Dyad_ = 10, *n*_minority_ = 5, *U* = 14, *p* = 0.206, two-tailed, Fig. 3).

Our full model included both condition (MIN-I vs. Dyad) and frequency of observations as fixed effects (Table S1). In this model, frequency of observations refers to the number of observations that an individual had made prior to the trial when they first used their untrained method. Where an individual never used their untrained method, we used the number of observations made at the time of their final trial in their first hour of testing. In the full model, Condition was found to have a significant effect but number of observations did not. To explore whether frequency of observations might influence MIN-I differently to those in the Dyad condition, we also fitted a model with an interaction between number of observations and Condition. In this interaction model, neither the interaction of frequency of observations with Condition nor frequency of observations itself was found to have a significant effect on switching behaviour (Table S1). Consequently, we dropped both the interaction and fixed effect of number of observations from the final model. In the final model, Condition was found to have a significant effect on whether individual’s switched to their untrained method on a given trial (Table 4), with an estimated probability that MIN-I would switch on a given trial of 0.518 (95% CI 0.447, 1) and a probability that individuals in the Dyad condition would switch of less than 0.001 (95% CI 0.000, 0.003). The proportion of variance explained by the fixed effects in the full model was *R*^2^ = 0.572.

## Discussion

Results showed that minority individuals (MIN-I) were, relative to majority individuals (MAJ-I), highly likely to switch from a pre-existing method to a socially demonstrated alternative in a group context, even though neither method was more efficient or productive. Moreover, MIN-I who switched methods did so rapidly (within first 5 trials, Table 2) and for three of those four individuals the change in behavioural preference remained relatively stable over time in group contexts (Fig. 2a). Conversely, most MAJ-I (all but three) faced with demonstrations of an alternative, equally rewarding behaviour from a lone minority did not switch method (Fig. 2b). Most individuals did not deviate from a pre-existing method when exposed to the alternative method of a single conspecific, nor did individuals who received no social information independently discover the alternative method. Crucially, the evidence does not support the possibility that switching was influenced by the frequency of observations (Fig. 3b), nor the number of individuals observed using the alternative method (Table 2, Table 4). Indeed, one MIN-I (‘HAN’) switched after observing just a handful of trials of a single individual. Because MIN-I were not aware that the observed method was preferred by the majority at the time of switching, this outcome does not easily align with conventional definitions of conformity. Due to the open-diffusion paradigm used, we were unable to systematically test each MIN-I after each additional group member was observed. This could be achieved in future using a more controlled design such as that used in Haun et al. (2014), but this is not straightforward in open-diffusion paradigms whose purpose is to simulate more naturalistic learning contexts. Consequently, it is not possible from the Group condition alone to determine whether all MIN-I who switched methods would, like ‘HAN’, have done so after observing just a single individual.

These results suggest that knowledgeable chimpanzees behave in a largely conservative manner unless they find themselves in a group context. Within a group context, most individuals switched to a consistently demonstrated behaviour. This outcome contrasts with prior work where it was found that individuals were likely to behave in a conservative manner (van Leeuwen et al. 2013; Haun et al. 2014; Vale et al. 2017). Our study differed methodologically from Haun et al. (2014) and Vale et al. (2017) in that our subjects were naïve to the untrained method before the Group condition, whereas in these earlier studies the behaviour may have acquired a negative valence through being previously experienced as ineffective (Haun et al. 2014) or associated with a less palatable food reward (Vale et al. 2017). Such was not the case in van Leeuwen et al. (2013), but in this case the trained behaviour was demonstrated by multiple group mates, potentially providing social reinforcement for the behaviour before subjects observed the untrained method. Moreover, changes in behavioural preference elicited in this context had a rapid onset (Table 2) and were stable over time, as MIN-I demonstrated behaviour consistent with their performance in the initial experimental period in an additional group session carried out 3 weeks afterwards (see Fig. 3). The importance of a group context in eliciting the use of an observed method is further reflected in the fact that there was no statistically significant repeatability in switching behaviour between the open-diffusion social context of Stage 2 and the solo context of Stage 3. This trend then reversed when individuals were put back into a group context for Stage 4, which had high repeatability with Stage 2. However, it is worth noting that there was substantial individual variation in how individuals behaved during the solo context of Stage 3, with one individual (HAN) retaining a strong preference for their untrained method, two others (AHN, IDA), gradually switching back to their trained method, one (DAH) showing a strong preference for their trained method and another (JOS) using their untrained method for the first time (Table 3).

In sum, our results suggest that being in a group context elicited the prioritisation of a very limited number of observations of a demonstrated method over well-established, pre-existing behaviours. This is somewhat surprising given that previous research has suggested that chimpanzees are highly conservative with regards to adopting novel behaviours. We suggest that social context is therefore a largely unexplored but potentially potent influence on behavioural flexibility and social learning that is worthy of further attention.

One way in which the group context may have elicited changes in behavioural preference is the possibility that MIN-I made inferences about the rest of the present group’s behavioural preferences based on their observations of a subset of individuals, and acted in a conformist fashion in accordance with this prediction. A similar proclivity to converge on perceived social norms, estimated from limited personal experience, has been established in humans (Rimal and Real 2005; Terry et al. 1999), and this capacity to generalise from small samples to a wider population is present in human infants as young as 8 months old (Denison and Xu 2010; Téglás et al. 2007; Xu and Garcia 2008). While all four great ape species have been shown to generalise from populations to samples (Rakoczy et al. 2014), evidence for inferences from samples to populations is somewhat mixed (Eckert et al. 2017). The phenomenon we have identified might correspond with ‘quorum sensing’, defined by Sumpter and Pratt (2009) as when ‘threshold group sizes trigger key changes in behaviour’. Although to our knowledge studies explicitly focused on this topic have hitherto been conducted on only insects (e.g. Pratt 2005) and fish (Ward et al. 2012), our results suggest more attention to such phenomena in primates and other vertebrates may prove fruitful.

Due to our study sharing similar behavioural outcomes to conformity, alternative explanations levelled at studies reporting this phenomenon (Acerbi et al. 2016; Van Leeuwen and Haun 2013, 2014; Van Leeuwen et al. 2015) should also be considered in relation to our results. For example, that individuals may ‘copy when uncertain’, such as when moving to a novel environment, is one alternative explanation offered (Van Leeuwen et al. 2015) for behavioural convergence in great tits (Aplin et al. 2015b) and vervet monkeys (van de Waal et al. 2013). In the case of our study, there were no such environmental changes and therefore no obvious reason for uncertainty-triggered copying. If through some mistake of design, some unintentional uncertainty was introduced by the paradigm, we would expect to see individuals in the Dyad condition also prioritise social information over their pre-existing method, but this was not the case. It has also been suggested that randomly copying a single individual could create an illusion of conformity to the options demonstrated by a majority in a group (Acerbi et al. 2016; Van Leeuwen et al. 2015). However, much previous research in chimpanzees has reported conservatism rather any evidence of random copying of observed methods (Davis et al. 2016; Haun et al. 2014; Hrubesch et al. 2009; Van Leeuwen et al. 2013). More importantly, random copying is not consistent with the outcome of our Dyad condition, where there was only a single individual to choose from, yet all but one individual remained faithful to their trained method.

A bias towards copying dominant individuals (‘rank bias’) is thought to influence from whom naïve chimpanzees choose to learn (Horner et al. 2010; Kendal et al. 2015) and therefore could also conceivably influence the social learning decisions of knowledgeable individuals. This would fit the pattern of results observed in the Group condition, as MIN-I were all judged as being medium to low in social rank by care staff and were therefore exposed to higher-ranking demonstrators than themselves. While we cannot rule this out entirely, it would be inconsistent with the results of the Dyad condition. Linear rank assessments for the Dyad condition were not practical, but rank disparities were inevitable due to the linear hierarchy of chimpanzee social structure. However, no individuals in the Dyad condition adopted their partner’s method. Moreover, studies in which a proportion of individuals with pre-existing behaviours were faced with more prestigious or more dominant demonstrators of the alternative method did not find evidence of behavioural switching (Haun et al. 2014; Van Leeuwen et al. 2013). This suggests that while *naïve* chimpanzees may selectively copy dominant models (Horner et al. 2010; Kendal et al. 2015, but see Watson et al. 2017), chimpanzees with an established method will not forego this in order to converge on the behaviour of these individuals.

We would emphasise, however, that while various social learning biases are often treated as competing explanations for the emergence of traditions, it is possible that they act in complementary ways and that different individuals make use of different learning strategies depending on their own life history. For example, while all children preferentially copy competent models, some prefer to copy a majority when given the choice (Burdett et al. 2016). It seems likely that similar variation could exist within the social learning habits of non-human species, and the individual differences that may contribute towards such variation will continue to confound studies of unitary biases until research on combinatorial effects is pursued. Our results are suggestive of notable individual differences in behavioural social information use. Most MIN-I rapidly converged on behaviours observed in a group context, while others did not, even after extensive exposure to demonstrations of the alternative method from a large number of individuals. Indeed, social learning biases are expected to vary between individuals within a species, since different phenotypic and life history factors may differently benefit from a given bias (Mesoudi et al. 2016). For example, our selection criteria for MIN-I that they had to be female low-to-middle social rank may have inadvertently selected for a class of individuals who tend to have a particularly high proclivity for social information. A way to control for this in future designs would be to use the same individuals in both Group and Dyad conditions—using different, but similar, puzzle boxes for each condition. This repeated-measures design would help minimise the confound of individual differences and isolate the influence of context. Our findings are broadly comparable with human conformity studies where, while *many* individuals conform to unanimous majorities, *most* do not (Asch 1951). Given the fission–fusion social structure that typifies wild chimpanzees, in the wild, individuals will often have information on the preferences of only a small sample of the larger community to which they belong, so being prepared to make inferences from a small sample to a larger group may be an adaptive strategy for them. They may also need to be somewhat flexible in terms of who they are motivated to socially learn from. Accordingly, we suggest that in future work additional attention be placed on the individual characteristics of the learners, as well as the models, and the contextual factors that may promote or inhibit ‘risky’ behaviour, such as exploring new methods (Davis et al. 2016). The use of methodologies that discriminate between multiple social learning biases (e.g. Kendal et al. 2015) operating at once in a single group, or a single individual, may be crucial.

Amongst the factors influencing social learning to be investigated in the future, based on our findings we strongly encourage the examination of how the presence and size of a group may influence copying decisions of animals. Our findings suggest that chimpanzees are more likely to relinquish existing behaviours in the presence of a social group, resulting in a convergence on an equally rewarding, socially demonstrated alternative. We submit this as a potential commonality between human and non-human animals in the potency of social influence, with important implications for the study of cultural diffusion.

## Electronic supplementary material

Below is the link to the electronic supplementary material.

Supplementary material 1 (DOCX 16 kb)
