# Supplementary material for: Chimpanzees prioritise social information over pre-existing behaviours in a group context but not in dyads
Source: Anim Cogn. 2018 Mar 24;21(3):407–18. doi: 10.1007/s10071-018-1178-y (PMC5908815; doi:10.1007/s10071-018-1178-y)
Supplement: Supplementary file 1 — Supplementary material 1 (DOCX 16 kb) [file 10071_2018_1178_MOESM1_ESM.docx]

| **Analysis** | **Model** | **Log likelihood** | **Fixed effect** | **Beta** | **Standard Error** | **Lower 95% CI** | **Upper 95% CI** | **Z** | **p** |
| --- | --- | --- | --- | --- | --- | --- | --- | --- | --- |
| 1 | Null | -358.2 | Intercept | -12.899 | 2.371 | -17.545 | -8.252 | -5.441 | - |
|  | Full | 352.7 | Intercept | -10.635 | 2.243 | -15.032 | -6.238 | -4.740 | - |
|  |  |  | Condition | 11.691 | 2.955 | 5.899 | 17.484 | 3.956 | <0.001 |
|  |  |  | Observations | -0.014 | 0.009 | -0.032 | 0.003 | -1.529 | 0.126 |
|  | Final | -354.0 | Intercept | -10.778 | 2.215 | -15.120 | -6.436 | -4.866 | - |
|  |  |  | Condition | 10.926 | 2.820 | 5.399 | 16.452 | 3.875 | <0.001 |
| 2 | Null | -132.6 | Intercept | -10.954 | 2.307 | -15.476 | -6.431 | -4.748 | - |
|  | Full | -127.3 | Intercept | -10.289 | 3.444 | -17.039 | -3.538 | -2.987 | - |
|  |  |  | Condition | 0.098 | 0.127 | 3.785 | 16.939 | 0.773 | 0.913 |
|  |  |  | Observation | 10.362 | 3.355 | -0.151 | 0.348 | 3.088 | 0.002 |
|  | Interaction | -126.8 | Intercept | -9.150 | 2.801 | -14.641 | -3.661 | -3.267 | - |
|  |  |  | Condition | 0.027 | 0.135 | 2.656 | 14.810 | 0.201 | 0.004 |
|  |  |  | Observation | 8.733 | 3.101 | -0.237 | 0.292 | 2.817 | 0.840 |
|  |  |  | Observation * Condition | 0.247 | 0.256 | -0.255 | 0.749 | 0.963 | 0.336 |
|  | Final | -127.7 | Intercept | -8.562 | 1.897 | 12.279 | 4.844 | -4.514 | - |
|  |  |  | Condition | 8.948 | 2.242 | 4.554 | 13.342 | 3.991 | <0.001 |
| 3 | Null | -18.0 | Intercept | -11.298 | 3.184 | -17.537 | -5.057 | -3.549 | - |
|  | Final | -18.0 | Intercept | 11.457 | 3.632 | -18.475 | -4.338 | -3.155 | - |
|  |  |  | Condition | 0.377 | 3.469 | -6.422 | 7.176 | 0.109 | 0.913 |

Table S1. Full outputs for each model tested in each analysis.
